# ZRSR1 protects against obesity-associated hepatic lipogenesis independent of minor intron splicing

**DOI:** 10.1093/lifemeta/loag020

**Published:** 2026-07-08

**Authors:** Yinkun Fu, Hongyong Song, Yinglin Han, Ding Chen, Wenyan Li, Ying Sun, Ming He, Xin Peng, Xu-Yun Zhao

**Affiliations:** Department of Biochemistry and Molecular Cell Biology, Shanghai Key Laboratory for Tumor Microenvironment and Inflammation, Key Laboratory of Cell Differentiation and Apoptosis of National Ministry of Education, Shanghai Jiao Tong University School of Medicine, Shanghai 200025, China; Institute for Translational Medicine on Cell Fate and Disease, Shanghai Ninth People’s Hospital, Key Laboratory of Cell Differentiation and Apoptosis of National Ministry of Education, Department of Pathophysiology, Shanghai Jiao Tong University School of Medicine, Shanghai 200011, China; Department of Biochemistry and Molecular Cell Biology, Shanghai Key Laboratory for Tumor Microenvironment and Inflammation, Key Laboratory of Cell Differentiation and Apoptosis of National Ministry of Education, Shanghai Jiao Tong University School of Medicine, Shanghai 200025, China; Department of Biochemistry and Molecular Cell Biology, Shanghai Key Laboratory for Tumor Microenvironment and Inflammation, Key Laboratory of Cell Differentiation and Apoptosis of National Ministry of Education, Shanghai Jiao Tong University School of Medicine, Shanghai 200025, China; Department of Biochemistry and Molecular Cell Biology, Shanghai Key Laboratory for Tumor Microenvironment and Inflammation, Key Laboratory of Cell Differentiation and Apoptosis of National Ministry of Education, Shanghai Jiao Tong University School of Medicine, Shanghai 200025, China; Department of Biochemistry and Molecular Cell Biology, Shanghai Key Laboratory for Tumor Microenvironment and Inflammation, Key Laboratory of Cell Differentiation and Apoptosis of National Ministry of Education, Shanghai Jiao Tong University School of Medicine, Shanghai 200025, China; Ruijin Hospital Luwan Branch, Shanghai Jiao Tong University School of Medicine, Shanghai 200020, China; Institute for Translational Medicine on Cell Fate and Disease, Shanghai Ninth People’s Hospital, Key Laboratory of Cell Differentiation and Apoptosis of National Ministry of Education, Department of Pathophysiology, Shanghai Jiao Tong University School of Medicine, Shanghai 200011, China; Department of Pathology, The Affiliated Hospital of Youjiang Medical University for Nationalities, Baise, Guangxi 533000, China; Department of Biochemistry and Molecular Cell Biology, Shanghai Key Laboratory for Tumor Microenvironment and Inflammation, Key Laboratory of Cell Differentiation and Apoptosis of National Ministry of Education, Shanghai Jiao Tong University School of Medicine, Shanghai 200025, China; Ruijin Hospital Luwan Branch, Shanghai Jiao Tong University School of Medicine, Shanghai 200020, China; Department of Biochemistry and Molecular Cell Biology, Shanghai Key Laboratory for Tumor Microenvironment and Inflammation, Key Laboratory of Cell Differentiation and Apoptosis of National Ministry of Education, Shanghai Jiao Tong University School of Medicine, Shanghai 200025, China; Ruijin Hospital Luwan Branch, Shanghai Jiao Tong University School of Medicine, Shanghai 200020, China

**Keywords:** hepatic steatosis, minor intron splicing, LXR-SREBP1c axis

## Abstract

Minor intron retention resulting from dysregulation of minor intron splicing factors is an emerging risk factor for the metabolic dysfunction–associated steatotic liver disease (MASLD). However, whether impaired expression of these splicing factors exerts critical roles in MASLD progression independent of their minor intron splicing activity remains largely undefined. Here, we show that hepatic expression of the minor intron splicing factor ZRSR1 (zinc finger CCCH-type, RNA-binding motif, and serine/arginine rich 1) was significantly downregulated in mice fed a high-fat diet (HFD), while canonical minor intron splicing remained unperturbed. Gain- and loss-of-function analyses in liver tissue confirmed that hepatic ZRSR1 negatively modulated HFD-triggered obesity and insulin resistance, indicating that ZRSR1 restrained MASLD progression through a minor intron splicing–independent mechanism. Notably, ZRSR1 cell-autonomously suppressed liver X receptor (LXR) activation–induced *de novo* lipogenesis in both hepatocyte models and mouse models. Mechanistically, we demonstrate that ZRSR1 directly interacted with LXRα, and this physical interaction suppressed the binding activity of the LXRα/retinoid X receptor α (RXRα) complex toward the promoter regions of its target genes including the sterol regulatory element-binding protein 1c (*Srebp1c*), consequently repressing the transcription of *Srebp1c* and its downstream lipogenic genes. Collectively, these findings define a novel, splicing-independent function of the minor intron splicing factor ZRSR1 in mediating hepatic *de novo* lipogenesis via constraining the LXR−SREBP1c axis, which in turn mitigates MASLD progression. This work expands the functional repertoire of minor intron splicing factors beyond their classical splicing roles, providing new insights into the molecular mechanisms underlying hepatic fatty acid metabolism and MASLD pathogenesis.

## Introduction

Metabolic dysfunction–associated steatotic liver disease (MASLD) represents a major and growing global health burden, with a current worldwide prevalence of approximately 30% [1, 2]. MASLD encompasses a spectrum of liver pathologies, ranging from simple hepatic steatosis to steatohepatitis, termed metabolic dysfunction-associated steatohepatitis (MASH), a condition that can progress to liver fibrosis, cirrhosis, and hepatocellular carcinoma (HCC) [3–5]. Importantly, MASLD is strongly associated with hypertriglyceridemia, glucose intolerance, insulin resistance, and type 2 diabetes mellitus, collectively making it an increasingly prominent global health challenge [6]. Despite its substantial clinical impact, the underlying molecular mechanisms of MASLD remain incompletely elucidated, and effective pharmacological therapies are still lacking [7, 8].

Minor introns, also termed U12-type introns, are rare yet physiologically critical components of eukaryotic genomes, accounting for only about 0.3% of all introns in humans but exhibiting high evolutionary conservation across species, underscoring their non-redundant functions [9–12]. The accurate splicing of these minor introns is strictly dependent on the minor spliceosome, a specialized ribonucleoprotein complex that localizes to the nucleus [13, 14]. As the dedicated machinery for excising U12-type introns, the minor spliceosome is orchestrated by a suite of highly conserved core factors, with ZRSR1 (zinc finger CCCH-type, RNA-binding motif, and serine/arginine rich 1) and ZRSR2 being pivotal components that mediate the recognition of U12-type intron sequences and facilitate the sequential splicing reactions [15, 16]. In humans, ZRSR2 serves as the primary minor intron splicing factor, given that *ZRSR1* is classified as a pseudogene; by contrast, both ZRSR1 and ZRSR2 are essential for minor intron splicing in mice [17]. Mutations or dysregulated expression of *ZRSR1* and *ZRSR2* lead to aberrant U12-type intron retention in target mRNAs, a defect that has been robustly linked to a broad range of human pathologies including developmental disorders, progressive neurodegenerative diseases, and various malignancies [18–21].

We previously demonstrated that hepatic deficiency of ZRSR1 and ZRSR2 triggers minor intron retention in insulin-induced gene 1 (*Insig1*) and *Insig2*, thereby inducing proteolytic activation of sterol regulatory element-binding protein 1c (SREBP1c) and promoting the progression of MASH [22]. However, the specific time point during MASLD development at which this minor intron retention initiates remains unexplored. In addition, whether these nuclear-localized minor intron splicing factors can transcriptionally regulate hepatic lipid metabolism independent of their essential role in U12-type intron splicing remains elusive.

From a well-established perspective, aberrant upregulation of hepatic *de novo* lipogenesis is a central driver of hepatic steatosis development, a process particularly pronounced in the setting of obesity and insulin resistance [23]. SREBP1c acts as a master transcriptional regulator of lipogenic genes, including stearoyl-CoA desaturase 1 (*Scd1*) and fatty acid synthase (*Fasn*) [24, 25]. As a nuclear receptor, liver X receptor (LXR) forms a transcriptional complex with retinoid X receptor (RXR) that directly binds to and activates the *Srebp1c* promoter, thereby upregulating the expression of *Srebp1c* and its downstream lipogenic genes [26–28]. Consequently, pharmacological activation of LXR by agonists such as T0901317 induces profound hepatic steatosis and hypertriglyceridemia, highlighting the pivotal role of the LXR−SREBP1c axis in hepatic lipid homeostasis.

In this study, we demonstrated that hepatic expression of *Zrsr1*, but not *Zrsr2*, is significantly downregulated in mice with obesity-associated hepatic steatosis. This reduction in ZRSR1 alone did not induce minor intron retention. Notably, liver-specific overexpression of *Zrsr1* alleviated MASLD ­progression and improved insulin resistance via a minor intron splicing-independent pathway, whereas hepatic *Zrsr1* knockout exerted the opposite effects. Furthermore, ZRSR1 cell-autonomously repressed LXR-driven hepatic *de novo* lipogenesis. Mechanistically, ZRSR1 physically interacted with LXRα, which weakened the promoter-binding ability of the LXRα-RXRα heterodimer. This event further inhibited *Srebp1c* transcription and consequently downregulated its downstream lipogenic genes. Therefore, our findings unveil a non-canonical function of the minor intron splicing factor ZRSR1 in directly repressing the lipogenic program, providing new insights into the complex regulation of hepatic lipid metabolism and identifying potential avenues for therapeutic intervention in MASLD.

## Results

### *Zrsr1* expression is downregulated in obesity-associated steatotic liver, yet such reduction does not induce obvious minor intron retention

**Figure 1:**
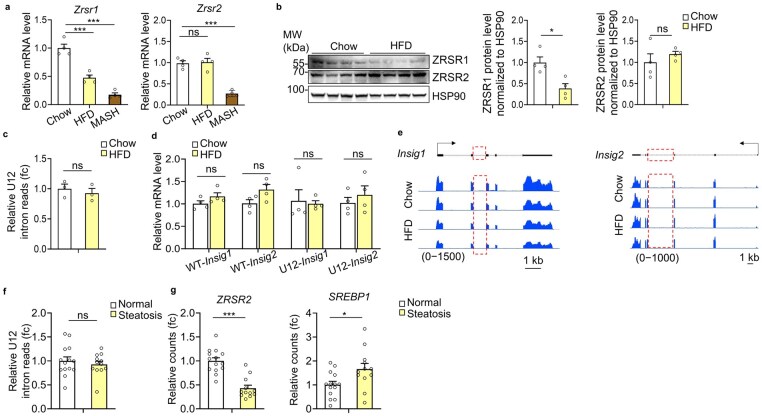
*Zrsr1* expression is downregulated in obesity-associated steatotic liver, yet such reduction does not induce obvious minor intron retention. (a and b) The qPCR (a) and immunoblotting (b) analyses of *Zrsr1* and *Zrsr2* expression in the livers of mice fed a chow diet (*n *= 4) as controls, an HFD (*n *= 4) for 4 months, or a CDA-HFD (MASH diet, *n *= 4) for 6 months. The relative amounts of ZRSR1 and ZRSR2 in b (left panel) were determined by the density of the corresponding bands of protein, normalized to that of HSP90. Then, the fold changes were calculated by dividing the relative protein density in livers from HFD-fed mice by that of chow diet-fed mice (b, right panel). (c) Fold change in reads mapping to minor (U12) introns from RNA-seq of livers from the chow diet- and HFD-fed mice, based on the IAOD. (d) The qPCR analyses of WT-*Insig1*, WT-*insig2*, minor intron (U12)-*Insig1*, and U12-*Insig2* in the livers of indicated mice. (e) Genome browser tracks showing peaks corresponding to U12 introns in *Insig1* and *Insig2* from the livers of chow diet- and HFD-fed mice. (f) Fold change in reads mapping to minor (U12) introns from RNA-seq of livers from the normal (*n *= 14) and steatotic patients (*n *= 12, GSE126848), based on IAOD. (g) Fold change in reads of *ZRSR2* and *SREBP1* from RNA-seq of livers from the normal and steatotic patients. Data are presented as the mean ± SEM. *P < 0.05; **P < 0.01; ***P < 0.001, by two-tailed unpaired Student’s *t*-test (b–d, f–g); by one-way ANOVA with Dunnett’s multiple comparisons test (a); ns, not significant.

MASLD is a disease spectrum that typically progresses from obesity-associated fatty liver to hepatitis and fibrosis. Our previous study demonstrated that downregulation of the minor intron splicing factors ZRSR1 and ZRSR2 in the livers of mice with MASH causes minor intron retention in *Insig* genes. This retention leads to proteolytic activation of SREBP1c and drives MASH progression [22], indicating that minor intron splicing defects are critical risk factors for MASLD. However, the specific time point during MASLD development at which minor intron retention initiates remain poorly understood. To clarify the time frame of minor intron splicing disruption in the early stages of MASLD progression, we investigated the expression of *Zrsr1* and *Zrsr2*, comparing their levels between the obesity-associated hepatic steatosis stage and the subsequent hepatic fibrotic stage of liver disease. Wild-type mice were fed a high-fat diet (HFD) for 4 months, with chow diet–fed mice serving as controls. After the feeding period, HFD-fed mice developed obesity and a fatty liver phenotype, whereas hepatic fibrosis was not observed (Supplementary Fig. S1). Quantitative PCR (qPCR) analysis revealed that hepatic *Zrsr1* expression was attenuated in HFD-fed mice, while *Zrsr2* expression remained unchanged. In contrast, the hepatic expression of both *Zrsr1* and *Zrsr2* was downregulated in mice with MASH as we previously reported (Fig. 1a). Consistently, immunoblotting results showed lower ZRSR1 protein levels in the livers of HFD-fed mice, whereas ZRSR2 was unaltered (Fig. 1b). ZRSR1 and ZRSR2 are homologous minor intron splicing factors in mice with compensatory functions. To determine whether downregulation of ZRSR1 alone is sufficient to induce global minor intron retention, we analyzed total minor intron reads according to the Intron Annotation and Orthology Database (IAOD) [12]. The results showed no significant changes in the fold change of minor (U12) intron reads in the livers between chow diet-fed and HFD-fed mice (Fig. 1c), indicating the absence of global minor intron retention. In our previous study, we demonstrated that attenuation of both ZRSR1 and ZRSR2 in the liver during the MASH stage induced minor intron retention in *Insig1* and *Insig2*. Thus, we measured the mRNA levels and minor intron retention levels of *Insig1* and *Insig2* in the livers of HFD-fed mice. Compared with chow diet–fed controls, neither the mRNA levels nor the minor intron retention levels of *Insig1* and *Insig2* showed significant alteration (fold change < 1.25) in HFD-fed mice (Fig. 1d). More notably, peak signals in the minor intron regions of *Insig1* and *Insig2* showed no retention in HFD-fed mice relative to controls (Fig. 1e). To validate the clinical relevance of impaired minor intron splicing factors in the hepatic steatosis stage, we analyzed an RNA sequencing (RNA-seq) dataset derived from human normal and steatotic liver tissues (GSE126848). Consistent with our murine model data, hepatic steatosis in humans did not induce minor intron retention (Fig. 1f). Furthermore, human *ZRSR2* expression was downregulated, and this regulatory pattern correlated with elevated *SREBP1* expression in these human samples (Fig. 1g). Collectively, these results indicate that downregulation of minor intron splicing factors commences during the obesity-associated hepatic steatosis stage of MASLD; however, this reduction is insufficient to elicit minor intron retention at this stage. This suggests that minor intron retention requires a profound reduction in minor intron splicing factor levels and likely initiates during the progression from steatosis to MASH.

### Hepatic *Zrsr1* expression ameliorates diet-induced obesity and insulin resistance

**Figure 2:**
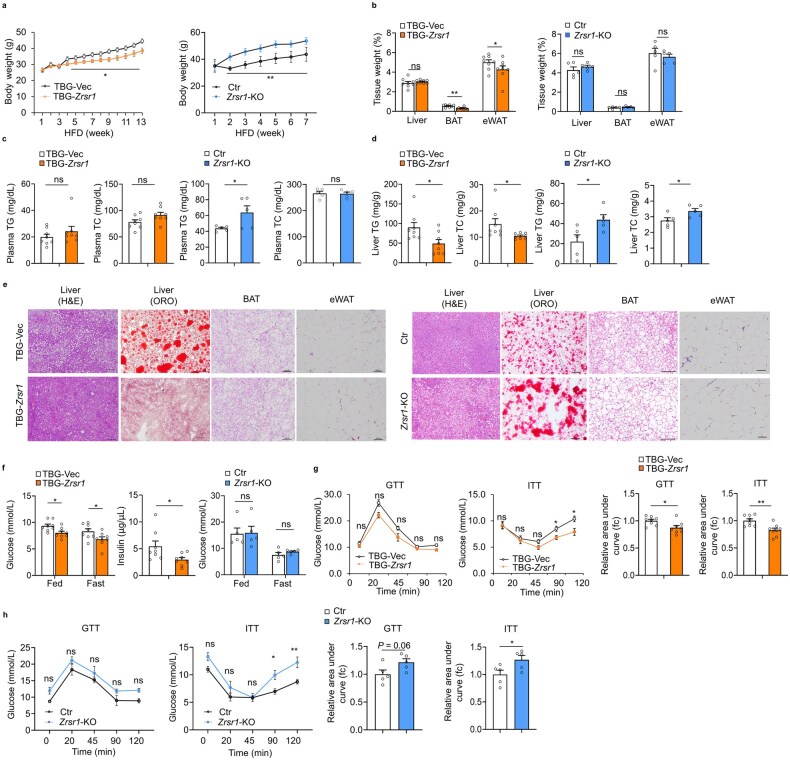
The expression of *Zrsr1* negatively modulates diet-induced obesity and insulin resistance. (a) Body weight curves of AAV-TBG-Vec (TBG-Vec, *n *= 8) and AAV-TBG-*Zrsr1* (TBG-*Zrsr1*, *n *= 8) mice (left) fed an HFD for 13 weeks, and of liver-specific Cas9 transgenic mice (right) injected with AAV expressing *Zrsr1*-targeting sgRNA (*Zrsr1*-KO, *n *= 5) or vector control (Ctr, *n *= 5) fed an HFD for 7 weeks. (b) Relative weights of liver, brown adipose tissue (BAT), and epididymal white adipose tissue (eWAT) (as percentages of body weight) in TBG-Vec and TBG-*Zrsr1* mice (left), and in hepatic *Zrsr1*-KO mice and controls (right) after HFD feeding. (c and d) Plasma (c) and liver (d) TG and TC levels of TBG-Vec and TBG-*Zrsr1* mice (left), and of hepatic *Zrsr1*-KO mice and controls (right) after HFD feeding. (e) H&E staining of liver and fat tissue, as well as Oil Red O (ORO) staining of livers from TBG-Vec and TBG-*Zrsr1* mice (left), and of hepatic *Zrsr1*-KO mice and controls (right) after HFD feeding (scale bar = 100 µm). (f) Plasma glucose levels under fasting or fed conditions (left), plasma insulin levels in fed conditions (middle) of TBG-Vec and TBG-*Zrsr1* mice, and blood glucose levels under fasting or fed conditions of hepatic *Zrsr1*-KO mice and controls (right) after HFD feeding. (g and h) GTT and ITT assays in the TBG-Vec and TBG-*Zrsr1* mice (g) and in hepatic *Zrsr1*-KO and control mice (h) after HFD feeding. The fold changes of area under the curves were calculated for both the GTT and ITT plots. Data are presented as the mean ± SEM. *P < 0.05; **P < 0.01; ***P < 0.001, by two-tailed unpaired Student’s *t*-test (b, c, d, f, and right panels of g and h); by two-way ANOVA with multiple comparisons (a and left panel of g and h); ns, not significant.

ZRSR1 is well-characterized as a splicing factor that mediates minor intron splicing during transcription. However, whether it exerts a minor intron splicing−independent role in the deve­lopment of MASLD remains uninvestigated. In our previous study, we depleted both *Zrsr1* and *Zrsr2* in mouse livers. Beyond inducing minor intron retention in *Insig* genes, which leads to SREBP1c cleavage, we also observed a significant upregulation of *Srebp1c* transcription in these mice. This finding suggests that ZRSR1 and ZRSR2 may suppress *Srebp1c* expression independently of their minor intron splicing activity. To further explore the minor intron splicing−independent role of ZRSR1 in the liver, we established liver-specific *Zrsr1*-overexpressing mice via intravenous injection of adeno-associated virus (AAV) vectors driven by the TBG promoter (Supplementary Fig. S2a and b). Meanwhile, we constructed hepatic *Zrsr1*-deficient mice by delivering *Zrsr1*-targeting sgRNA-containing AAV into liver-specific Cas9 transgenic mice (Supplementary Fig. S2c). Neither *Zrsr1*-overexpressing nor *Zrsr1*-knockout models led to global minor intron retention (Supplementary Fig. S2d). When fed a chow diet, both *Zrsr1*-overexpressing and *Zrsr1*-deficient mice exhibited body weight and liver weight comparable to those of control mice (Supplementary Fig. S2e and f). In addition, plasma levels of non-esterified fatty acids (NEFA), triglycerides, and cholesterol, as well as liver histology, remained unaltered (Supplementary Fig. S2g–i). We then fed these mice an HFD to investigate the role of ZRSR1 during the obesity-associated fatty liver stage. No significant differences were observed in food intake among these mice (Supplementary Fig. S2j). Interestingly, mice with hepatic *Zrsr1* overexpression exhibited attenuated body weight gain upon HFD challenge, accompanied by reduced mass of brown and white adipose tissues. Conversely, liver-specific *Zrsr1* knockout accelerated body weight gain (Fig. 2a and b). Notably, liver-specific *Zrsr1* overexpression markedly lowered hepatic triglyceride and cholesterol accumulation, while *Zrsr1* knockout produced the contrary phenotypes. Intriguingly, *Zrsr1* knockout also elevated circulating triglyceride levels while both plasma triglyceride and cholesterol remained unaffected in *Zrsr1*-overexpressing mice (Fig. 2c and d). Liver histological examination and Oil Red O (ORO) staining revealed that HFD-induced hepatic steatosis was prominently alleviated in *Zrsr1*-overexpressing mice but markedly exacerbated in *Zrsr1*-knockout mice, while the morphology of brown and white adipose tissue remained similar to that of controls (Fig. 2e). Obesity-associated hepatic steatosis is frequently accompanied by insulin resistance. We therefore assessed glucose levels and insulin sensitivity in these mice. Hepatic *Zrsr1* overexpression lowered the blood glucose levels in both fed and fasting states and the plasma insulin in the fed state, whereas *Zrsr1* knockout had no obvious effect on blood glucose levels (Fig. 2f). Glucose tolerance test (GTT) and insulin tolerance test (ITT) further verified that hepatic *Zrsr1* overexpression improved insulin sensitivity, whereas *Zrsr1* deficiency led to insulin resistance (Fig. 2g and h). Overall, these data indicate that hepatic ZRSR1 exerts a minor intron splicing−independent role in mitigating diet-induced obesity and insulin resistance.

### ZRSR1 attenuates hepatic *de novo* lipogenesis and *Srebp1c* expression

**Figure 3:**
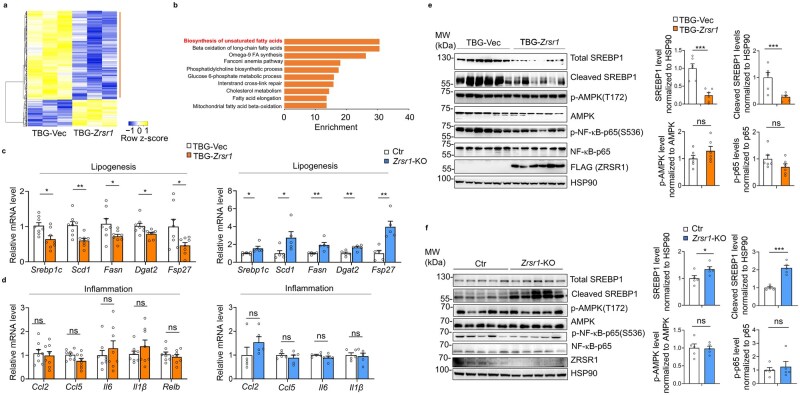
ZRSR1 attenuates hepatic *de novo* lipogenesis and *Srebp1c* expression. (a) Heatmap showing a set of genes whose expression is downregulated in the livers of AAV-TBG-*Zrsr1*–injected mice compared to those in AAV-TBG-Vec–injected mice fed an HFD. (b) Gene Ontology analysis showing enriched pathways associated with genes downregulated in hepatic *Zrsr1*-overexpression mouse livers. (c and d) The qPCR analyses of lipogenesis genes (c) and inflammation genes (d) in the livers of TBG-Vec (*n *= 8) and TBG-*Zrsr1* (*n *= 8) mice (left), and of hepatic *Zrsr1*-KO mice (*n *= 5) and controls (*n *= 5, right) fed an HFD. (e and f) Immunoblotting analyses of liver lysates from TBG-Vec and TBG-*Zrsr1* mice (e) and from hepatic *Zrsr1*-KO and control mice (f) fed an HFD. The relative amounts of proteins were determined by the density of the corresponding bands of protein, normalized to that of HSP90 (for total and cleaved SREBP1), AMPK (for p-AMPK), or p65 (for p-p65). Then, the fold changes were calculated by dividing the relative protein density in livers from TBG-*Zrsr1* mice by that of TBG-Vec mice (e, right panel) and hepatic *Zrsr1*-KO mice by that of controls (f, right panel). Data are presented as the mean ± SEM. *P < 0.05; **P < 0.01; ***P < 0.001, by two-tailed unpaired Student’s *t*-test (c–f); ns, not significant.

To explore the potential pathways regulated by *Zrsr1* overexpression, we performed RNA-seq on the livers of control and hepatic *Zrsr1*-overexpressing mice following HFD feeding. RNA-seq analysis showed that a large subset of genes was drastically downregu­lated, and enrichment analysis indicated that these genes were primarily involved in the biosynthesis of unsaturated fatty acids (Fig. 3a and b). The qPCR analysis revealed reduced hepatic expression of *de novo* lipogenesis-related genes in *Zrsr1*-overexpressing mice, including the transcription factor *Srebp1c*, its downstream enzymes *Scd1*, *Fasn*, and diacylglycerol acyltransferase 2 (*Dgat2*), as well as the lipid droplet marker fat-specific protein 27 (*Fsp27*) (Fig. 3c). Conversely, all these genes were significantly upregulated in the livers of *Zrsr1*-knockout mice (Fig. 3c). Interestingly, the expression levels of inflammation-related genes showed no signifi­cant alterations in either *Zrsr1*-overexpressing or *Zrsr1*-knockout mice (Fig. 3d). Western blot results further validated that the protein levels of both precursor and mature cleaved SREBP1 were reduced in the livers of HFD-fed *Zrsr1*-overexpressing mice, while these proteins were obviously enhanced in *Zrsr1*-knockout mice. Neither the phosphorylation nor the total protein level of adenosine 5’-monophosphate-activated protein kinase (AMPK), a key regulator of *de novo* lipogenesis, was affected (Fig. 3e and f). Meanwhile, phosphorylation of the immune-associated nuclear factor kappa-B (NF-κB) p65 subunit was unaltered, indicating that inflammation was not affected (Fig. 3e and f). Collectively, these data suggest that hepatic ZRSR1 suppresses *de novo* lipogenesis, likely by attenuating the expression of *de novo* lipogenic genes.

### ZRSR1 restricts LXR activation−induced *de novo* lipogenesis in mouse liver

**Figure 4:**
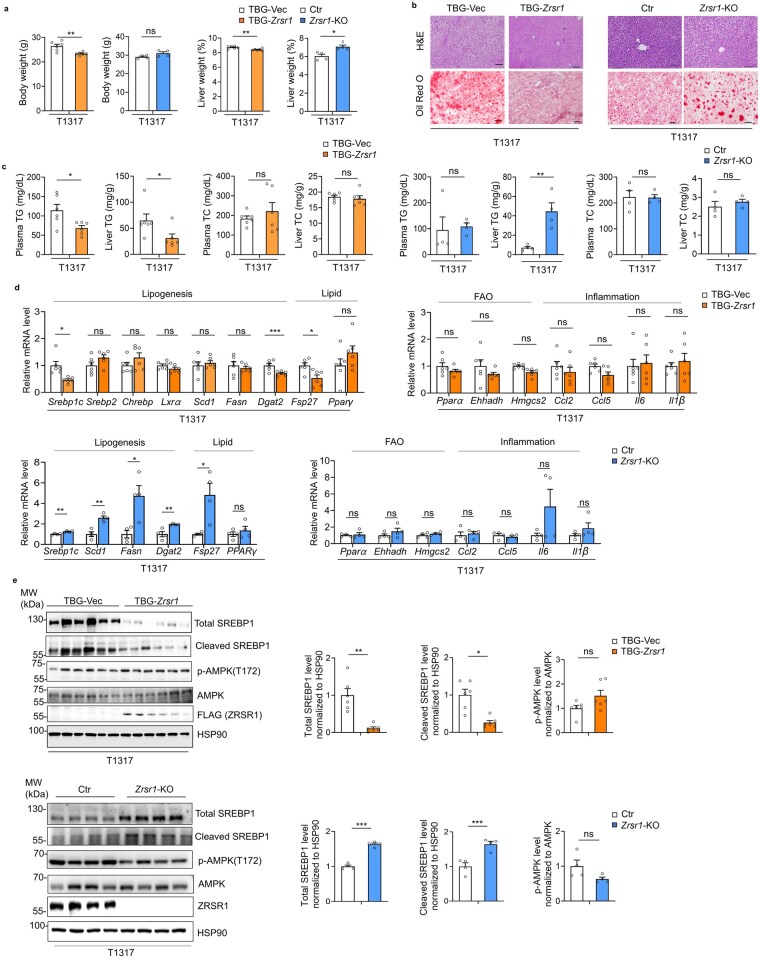
ZRSR1 restricts LXR activation−induced *de novo* lipogenesis in mouse liver. (a) Body weight (left two panels) and relative liver weight (percentage of body weight, right two panels) of AAV-TBG-vector (TBG-Vec, *n *= 6) and AAV-TBG-*Zrsr1* (TBG-*Zrsr1*, *n *= 6) mice fed an HFD, followed by oral gavage of LXR agonist T0901317 (T1317, 25 mg/kg/day) for 4 consecutive days, and of hepatic *Zrsr1*-KO (*n *= 4) and control mice (*n *= 4), followed by oral gavage of T1317 for 3 consecutive days. (b) H&E staining and ORO staining of livers from TBG-Vec and TBG-*Zrsr1* mice (left), and from hepatic *Zrsr1*-KO and control mice (right) after T1317 treatment (scale bar = 100 µm). (c) Plasma and liver contents of TG and TC in TBG-Vec and TBG-*Zrsr1* mice (left four panels), and in hepatic *Zrsr1*-KO and control mice (right four panels) treated with T1317. (d) The qPCR analysis of genes involved in lipogenesis, lipid metabolism, fatty acid oxidation, and inflammation in livers from TBG-Vec and TBG-*Zrsr1* mice (up panel), and from hepatic *Zrsr1*-KO and control mice (down panel), treated with T1317. (e) Immunoblotting analysis of liver lysates from TBG-Vec and TBG-*Zrsr1* mice (up panel), and from hepatic *Zrsr1*-KO and control mice (down panel), treated with T1317; The relative amounts of proteins were determined by the density of the corresponding bands of protein, normalized to that of HSP90 (for cleaved and total SREBP1) or AMPK (for p-AMPK). Then, the fold changes were calculated by dividing the relative protein density in livers from TBG-*Zrsr1* mice by that of TBG-Vec mice, or by dividing the relative protein density in livers from hepatic *Zrsr1*-KO mice by that of controls (the right of each immunoblot). Data are presented as the mean ± SEM. *P < 0.05; **P < 0.01; ***P < 0.001, by two-tailed unpaired Student’s *t*-test (a, c–e); ns, not significant.

The LXR−SREBP1c axis regulates *de novo* lipogenesis, and its aberrant activation is a key driver of hepatic steatosis development. To further investigate whether ZRSR1 inhibits LXR ­activation−driven hepatic lipogenesis *in vivo*, we administered the LXR agonist T0901317 (T1317) via gavage to control mice and mice with hepatic *Zrsr1* overexpression and knockout. Upon T1317 administration, *Zrsr1*-overexpressing mice displayed reduced body weight and liver weight relative to control counterparts, whereas *Zrsr1*-knockout mice exhibited no obvious changes in body weight, yet their liver weight was markedly increased (Fig. 4a). Hematoxylin and eosin (H&E) as well as ORO staining revealed that liver-specific *Zrsr1* overexpression alleviated hepatic lipid deposition and steatosis triggered by LXR activation, whereas *Zrsr1* deficiency aggravated these pathological phenotypes (Fig. 4b). Specifically, *Zrsr1* overexpression significantly reduced triglyceride contents in both the plasma and the liver, whereas cholesterol levels in the plasma and the liver remained unaffected. Consistently, *Zrsr1* knockout had higher hepatic triglyceride levels (Fig. 4c). The qPCR analysis showed that the mRNA levels of lipogenic genes including *Srebp1c*, *Dgat2*, and the lipid droplet marker *Fsp27* were significantly reduced in the livers from *Zrsr1*-overexpressing mice. Conversely, the mRNA levels of *Srebp1c*, *Fasn*, *Scd1, Dgat2,* and *Fsp27* were upregulated in the livers from *Zrsr1*-deficient mice. Notably, genes involved in fatty acid oxidation and inflammatory response in both groups remained unchanged (Fig. 4d). Western blot analysis further verified that upon LXR activation, both total and mature cleaved SREBP1 protein levels were markedly reduced in the livers from *Zrsr1*-overexpressing mice, yet significantly elevated in the livers from *Zrsr1*-knockout mice. Moreover, no obvious differences in total AMPK protein or its phosphorylation levels were detected between *Zrsr1*-overexpressing and control littermates, or between *Zrsr1*-knockout mice and control littermates (Fig. 4e). Collectively, these findings indicate that hepatic ZRSR1 serves as a critical negative regulator of LXR-driven *de novo* lipogenesis in the liver.

### ZRSR1 reduces LXR−SREBP1c axis activity in hepatocytes

**Figure 5:**
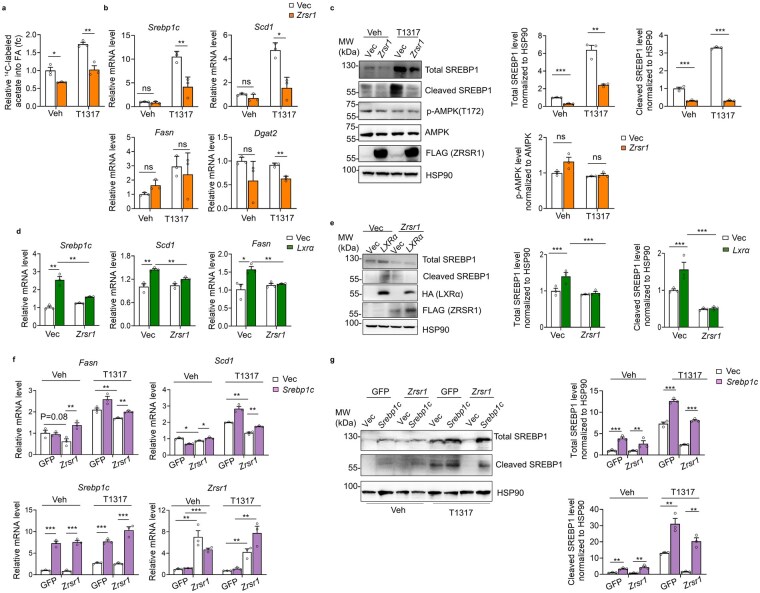
ZRSR1 reduces LXR−SREBP1c axis activity in hepatocytes. (a) *De novo* lipogenesis assay measuring the incorporation of [U-^14^C] acetate acid into lipids in Vec- and *Zrsr1*-overexpressing AML12 cells, treated with vehicle (Veh) or T1317 (5 µmol/L) for 24 h. (b) The qPCR analysis of hepatic genes involved in lipogenesis in Vec- and *Zrsr1*-overexpressing AML12 cells treated with Veh or T1317 (5 µmol/L, 24 h). (c) Immunoblotting analysis of cell lysates from Vec- and *Zrsr1*-overexpressing AML12 cells after vehicle (Veh) or T1317 treatment. The relative amounts of proteins were determined by the density of the corresponding bands of protein, normalized to that of HSP90 (for cleaved and total SREBP1) or AMPK (for p-AMPK). Then, the fold changes were calculated by dividing the relative protein density of AML12 cells expressing *Zrsr1* by that of AML12 cells expressing Vector (right panels). (d) The qPCR analysis of hepatic genes involved in lipogenesis in Vec- and *Lxrα*-overexpressing AML12 cells infected with Vec or *Zrsr1* adenovirus for 24 h. (e) Immunoblotting analysis of cell lysates from Vec- and *Lxrα*-overexpressing AML12 cells infected with Vec or *Zrsr1* adenovirus for 24 h. The relative amounts of proteins were determined by the density of the corresponding bands of protein, normalized to that of HSP90. Then, the fold changes were calculated by dividing the relative protein density of Vec- and *Lxrα*-overexpressing AML12 cells infected with *Zrsr1* adenovirus by that of cells infected with Vec adenovirus (right pa­nels). (f) The qPCR analysis of hepatic genes involved in lipogenesis in Vec- and *Srebp1c*-overexpressing AML12 cells infected with Vec or *Zrsr1* adenovirus and then treated with vehicle (Veh) or T1317 (5 µmol/L) for 24 h. (g) Immunoblotting analysis of cell lysates from Vec- and *Srebp1c*-overexpressing AML12 cells infected with Vec or *Zrsr1* adenovirus and then treated with Veh or T1317 for 24 h. The relative amounts of proteins were determined by the density of the corresponding bands of protein, normalized to that of HSP90. Then, the fold changes were calculated by dividing the relative protein density of Vec- and *Srebp1c*-overexpressing AML12 cells infected with *Zrsr1* adenovirus by that of cells infected with Vec adenovirus after T1317 or Veh treatment (right panels). Data are presented as the mean ± SEM. *P < 0.05; **P < 0.01; ***P < 0.001, by two-tailed unpaired Student’s *t*-test (a, b, and right panel of c); by one-way ANOVA with Dunnett’s multiple comparisons test (d, f, and right panels of e and g). ns, not significant.

To further validate that ZRSR1 suppresses *de novo* lipogenesis in hepatocytes, we performed a *de novo* lipogenesis assay using [U-^14^C]-labeled acetate as a tracer in the normal mouse hepatic cell line AML12. In addition, we treated cells with the LXR agonist T1317 to activate LXR signaling. The results showed that *Zrsr1* overexpression significantly suppressed *de novo* lipogenesis, as evidenced by reduced flux of [U-^14^C]-labeled acetate to fatty acids, an effect that was particularly pronounced upon LXR activation (Fig. 5a). The qPCR analysis revealed that LXR activation induced the expression of *de novo* lipogenesis genes including *Srebp1c* and *Scd1.* Notably, *Zrsr1* overexpression blunted this inductive effect (Fig. 5b). A similar inhibitory effect of ZRSR1 on lipogenic gene expression was observed in primary hepatocytes treated with T1317 (Supplementary Fig. S3). Consistent with these findings, western blot analysis confirmed that T1317 treatment increased the levels of precursor SREBP1 and its active form in AML12 cells. *Zrsr1* overexpression effectively abolished such upregulation. Notably, no significant changes in AMPK activation were observed (Fig. 5c). To further solidify the inhibitory role of ZRSR1 in the LXR activation−driven lipogenic program, we first overexpressed *Lxrα* in hepatocytes and subsequently introduced *Zrsr1* overexpression. *Lxrα* overexpression transcriptionally upregulated *de novo* lipogenic genes; however, qPCR results revealed that *Zrsr1* overexpression inhibited not only *Srebp1c* transcription but also the expression of its downstream lipogenic genes, *Scd1* and *Fasn*, even in the context of *Lxrα* overexpression (Fig. 5d). Western blot analysis further confirmed that co-expression of *Zrsr1* with *Lxrα* reduced both precursor and mature nuclear SREBP1 protein levels (Fig. 5e). Given that our results suggested that ZRSR1 may suppress LXR activation−driven lipogenesis by reducing *Srebp1c* transcription, and considering that SREBP1c is a master regulator controlling the entire lipogenic program, we next sought to confirm whether ZRSR1 suppresses *de novo* lipogenesis through inhibiting *Srebp1c* expression. To this end, we rescued *Srebp1c* expression in *Zrsr1*-overexpressing AML12 cells. Notably, *Srebp1c* overexpression significantly reversed the ZRSR1-mediated suppression of lipogenic gene expression (Fig. 5f and g). Taken together, these results demonstrate that ZRSR1 directly suppresses LXR−SREBP1c axis-induced *de novo* lipogenesis in hepatocytes.

### ZRSR1 suppresses *Srebp1c* transcription through interaction with LXRα

**Figure 6:**
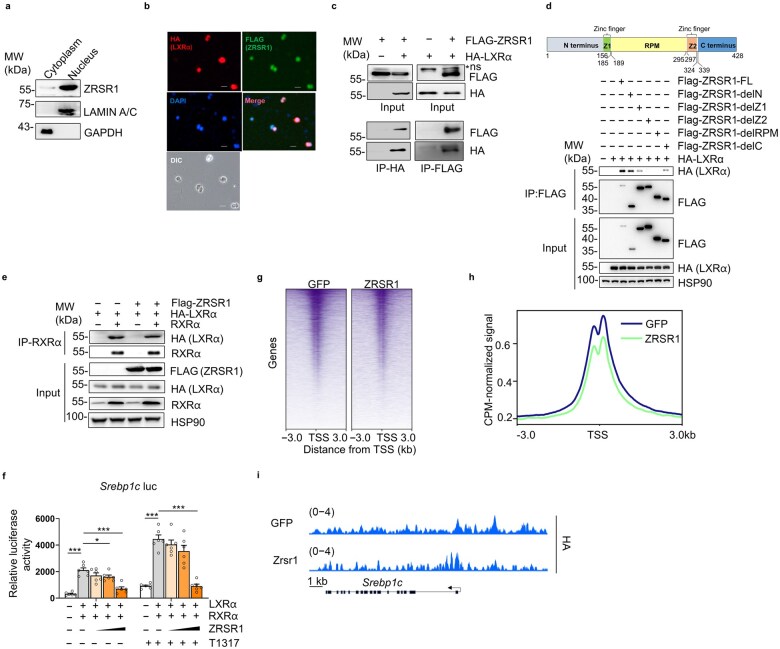
ZRSR1 suppresses *Srebp1c* transcription through interaction with LXRα. (a) Immunoblotting analysis showing the subcellular location of ZRSR1 in AML12 cells. LAMIN A/C and GAPDH serve as nuclear and cytoplasmic markers, respectively. (b) Immunofluorescence staining of HA-LXRα (red) and Flag-ZRSR1 (green) in 293 T cells co-transfected with HA-LXRα and Flag-ZRSR1-expressing plasmids; Scale bar = 20 μm. (c) Co-IP assays demonstrating the interaction between ZRSR1 and LXRα. HEK293T cells were transiently transfected with Vec or HA-LXRα together with Flag-ZRSR1. Cell lysates were subjected to IP with HA antibody (left panel). HEK293T cells were transiently transfected with Vec or Flag-ZRSR1 together with HA-LXRα, using an anti-Flag antibody for IP (right panel). (d) Diagram showing the ZRSR1 protein structure (up panel) and Co-IP assay determining the functional domain of ZRSR1 interacted with LXRα (down panel). HA-LXRα plasmid was co-transfected with a plasmid expressing full-length Flag-*Zrsr1* or various truncated mutants into 293 T cells, and Flag antibody was used for Co-IP to determine the functional domain of ZRSR1 interacted with LXRα (down panel). (e) Co-IP assays showing interactions between HA-LXRα and RXRα in 293 T cells transfected with or without Flag-ZRSR1, using an anti-RXRα antibody for IP. (f) Luciferase reporter assays using the *Srebp1c* promoter. Luciferase reporter assays were performed in AML12 cells co-transfected with *Srebp1c* promoter reporter plasmid and plasmids expressing LXRα and RXRα, which were infected with Vec or different doses of *Zrsr1* adenovirus and then treated with Veh or T1317. (g and h) Heatmap (g) and binding profile (h) showing genome-wide LXRα occupancy at genomic loci in *Zrsr1*- or GFP-overexpressing AML12 cells, characterized via Cut & Tag sequencing. (i) Cut & Tag sequencing determining LXRα occupancy at the *Srebp1c* promoter in *Zrsr1*- or GFP-overexpressing AML12 cells, visualized in the Genome Browser. Data are presented as the mean ± SEM. *P < 0.05; **P < 0.01; ***P < 0.001, by one-way ANOVA with Dunnett’s multiple comparisons test (f); ns, not significant.

The above results demonstrate that ZRSR1 inhibited hepatic *de novo* lipogenesis by downregulating LXR−SREBP1c axis activity, and this function ultimately mitigated diet-induced obesity and insulin resistance. Mechanistically, we hypothesized that ZRSR1, as a nuclear RNA-binding protein, directly associates with LXRα to modulate *Srebp1c* expression and the *de novo* lipogenic program. Subcellular fractionation assays confirmed the predominant nuclear localization of ZRSR1 (Fig. 6a), and immunofluorescence staining further revealed that ZRSR1 colocalized with LXRα in the nucleus (Fig. 6b). We next investigated the interaction between ZRSR1 and LXRα using co-immunoprecipitation (Co-IP) assays, which clearly demonstrated a physical interaction between ZRSR1 and LXRα (Fig. 6c). We then sought to identify the functional domains responsible for this interaction. Using a series of truncated *Zrsr1* mutants, we confirmed that the second zinc finger domain (Z2) and RNA binding domain were essential for the physical binding between ZRSR1 and LXRα (Fig. 6d). It is well established that LXRα and RXRα form a heterodimeric transcriptional complex that mediates the expression of *Srebp1c* and other lipogenic genes. Notably, the interaction between ZRSR1 and LXRα did not disrupt LXRα-RXRα heterodimer formation (Fig. 6e). Meanwhile, our cycloheximide chase assays revealed that *Zrsr1* overexpression exerted no obvious effects on LXRα protein stability (Supplementary Fig. S4a and b). To determine whether ZRSR1 influences the ability of the LXR/RXR complex to bind and activate the *Srebp1c* promoter, we performed luciferase reporter assays using the *Srebp1c* promoter. In AML12 cells, overexpression of *Lxrα/Rxrα* strongly induced *Srebp1c* promoter activity, an effect that was markedly suppressed by *Zrsr1* overexpression in a dose-dependent manner, both under basal conditions and upon stimulation with the LXR agonist T1317 (Fig. 6f). To further explore whether the interaction between ZRSR1 and LXRα modulates global genomic binding activity of the LXR/RXR complex to target gene promoters, we performed cleavage under targets & tagmentation (Cut & tag) assays to assess the effect of *Zrsr1* overexpression on LXRα chromatin occupancy. Our results revealed that *Zrsr1* overexpression weakened the binding affinity of LXRα to the promoter of target genes including *Srebp1c*, thus lowering its chromatin binding enrichment (Fig. 6g–i). These findings provide direct molecular evidence that ZRSR1−LXRα interaction impedes the binding activity of the LXR/RXR complex toward its downstream target genes.

Taken together, these findings support a working model wherein ZRSR1 localizes to the nucleus, interacts with LXRα, and represses *Srebp1c* transcription by interfering with the binding activity of the LXR/RXR complex to the *Srebp1c* promoter. This, in turn, downregulates lipogenic gene expression and attenuates *de novo* lipid synthesis, ultimately conferring protection against MASLD progression.

## Discussion

ZRSR1 is a core component of the minor spliceosome that mediates the splicing of U12-type minor introns, a highly conserved process essential for maintaining the integrity of transcripts encoding proteins critical to cellular physiological functions. Our previous work demonstrated that dysregulated ZRSR1 and ZRSR2 play a pivotal role in the progression of MASH [22]. Specifically, loss of these splicing factors in MASH leads to minor intron retention in *Insig1* and *Insig2*, which in turn triggers proteolytic activation of SREBP1c during the MASH phase. However, the precise timeframe for the onset of minor intron splicing defects during MASLD remains incompletely defined.

In this article, we used an HFD-induced obese mouse model to investigate minor intron splicing during hepatic steatosis, the initial stage of MASLD progression. Notably, we observed selective downregulation of hepatic ZRSR1, but not ZRSR2, in mice with HFD-induced hepatic steatosis. In addition, *ZRSR2* expression was also reduced in the liver tissues of human patients with hepatic steatosis. More importantly, the downregulation of minor intron splicing factors at this early stage of MASLD was insufficient to induce global minor intron retention or retention at the *Insig1* and *Insig2* loci. Therefore, these findings suggest a decoupling between the dysregulation of minor intron splicing factor expression and minor intron splicing deficiency. Specifically, the reduction in minor intron splicing factors, particularly ZRSR1, occurs at the early steatotic stage of MAFLD, whereas minor intron retention likely initiates during the progression from steatosis to MASH in the pathogenesis of MASLD.

More interestingly, our data demonstrated that ZRSR1 downregulation contributed to hepatic steatosis via a minor intron splicing−independent mechanism. To verify this, we established HFD-induced obese mice with liver-specific *Zrsr1* overexpression or knockout, in which minor intron retention was not induced. Intriguingly, *Zrsr1* overexpression not only significantly reduced obesity progression but also substantially decreased hepatic lipid deposition, as well as triglyceride and cholesterol levels. Consequently, insulin resistance in these mice was notably alleviated. Conversely, ZRSR1 deficiency accelerated the progression of obesity and MASLD. To further explore the mechanism by which ZRSR1 regulates hepatic lipid metabolism, we conducted RNA-seq analyses. Clustering results revealed that hepatic *de novo* lipoge­nesis was suppressed by *Zrsr1* overexpression. More importantly, the activation of LXR−SREBP1c axis, a key regulatory pathway for hepatic *de novo* lipogenesis, was downregulated by ZRSR1, and this was supported by multiple lines of evidence. First, hepatic *Zrsr1* overexpression significantly suppressed the transcriptional levels of lipogenic genes, including *Srebp1c* and its downstream lipogenic genes, *Fasn* and *Scd1*. At the protein level, both the abundance of precursor SREBP1 and the proteolytic activation of SREBP1 were inhibited. Opposite molecular alterations were observed in liver-specific *Zrsr1*-knockout mice. Second, using a mouse model treated with the LXR agonist T0901317, we demonstrated that hepatic *Zrsr1* overexpression in mice inhibited *de novo* lipogenesis upon LXR activation, as evidenced by reduced hepatic and plasma triglyceride levels, as well as decreased hepatic lipid deposition and lipogenic gene expression. In addition, in the normal hepatocyte cell line AML12 and primary mouse hepatocytes, *Zrsr1* overexpression effectively suppressed the transcriptional expression of *Srebp1c* under both LXR agonist-treated and LXR-overexpressed conditions. Reciprocal regulatory effects were validated in liver-specific *Zrsr1*-deficient mice. More compellingly, in AML12 cells, ZRSR1 exerted a suppressive effect on hepatic *de novo* lipogenic function, an effect confirmed by reduced incorporation of ^14^C-labeled acetate into fatty acids, a direct readout of *de novo* lipogenesis activity, under both basal and LXR-activated conditions.

To further verify that ZRSR1 suppresses the *de novo* lipogenic program primarily by disrupting LXR activation−induced *Srebp1c* expression, we restored *Srebp1c* expression in *Zrsr1*-overexpressing cells. Notably, this *Srebp1c* rescue largely restored the impaired lipogenic gene program in *Zrsr1*-overexpressing cells, even under LXR activation. Collectively, these findings indicate that ZRSR1 exerts a minor intron splicing−independent role in regulating the transcriptional expression of *Srebp1c* during hepatic steatosis, which is mediated by modulating the activity of the LXR−SREBP1c axis.

LXR and RXR form a transcriptional complex that directly binds to the *Srebp1c* promoter to induce *Srebp1c* expression. ZRSR1 localizes to the nucleus, where it participates in assembling the minor spliceosome, raising the possibility of a physical interaction between ZRSR1 and the LXR/RXR complex. In our study, we demonstrated that ZRSR1 physically interacts with LXRα. More importantly, this interaction enables ZRSR1 to dose-dependently inhibit the activity of the *Srebp1c* promoter driven by the LXR/RXR complex. Notably, ZRSR1 does not interfere with the assembly of the LXR/RXR heterodimer itself. Instead, it curtails the recruitment of this complex to target gene promoters and decreases the chromatin occupancy of LXRα. These findings indicate that ZRSR1 attenuates the binding of the LXR/RXR complex to target gene promoters, thereby suppressing the transcription of *Srebp1c*.

Overall, this study provides evidence that ZRSR1 exerts a minor intron−independent role in suppressing the activity of the LXR−SREBP1c axis, thereby expanding the known functions of minor intron splicing factors beyond their canonical roles in RNA splicing. Given that minor intron splicing disruption likely occurs during the transition from hepatic steatosis to MASH, the downregulation of minor intron splicing factors in the MASH stage may simultaneously induce *Srebp1c* transcription and SREBP1c cleavage via both minor intron splicing−dependent and −independent pathways. These findings suggest that minor intron splicing factors exert stage-specific functions during MASLD progression. At the hepatic steatosis stage, ZRSR1 downregulation promotes *Srebp1c* transcription by enhancing LXR−SREBP1c axis activity; at the hepatic fibrosis stage, more extensive downregulation of both ZRSR1 and ZRSR2 initiates minor intron retention, which in turn activates SREBP1c cleavage, thus upregulating *Srebp1c* expression at both the mRNA and protein levels.

**Figure 7:**
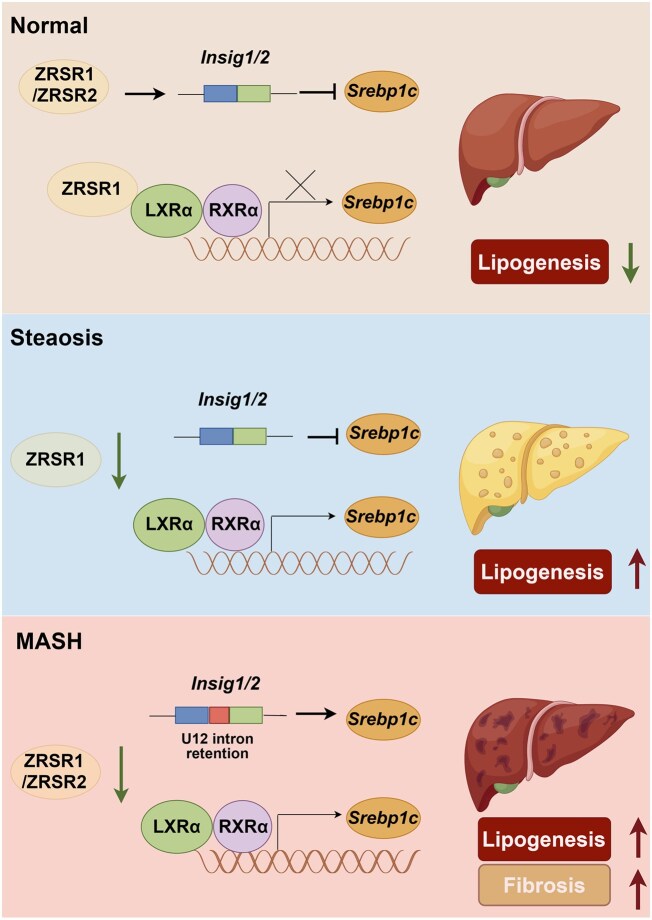
The stage-specific functions of minor intron splicing factors during MASLD progression. Schematic illustration shows the dynamic role of minor intron splicing factors during MASLD progression. At the hepatic steatosis stage, ZRSR1 downregulation promotes *Srebp1c* transcription via enhanced LXR−SREBP1c axis activity, whereas more extensive downregulation of both ZRSR1 and ZRSR2 at the MASH stage triggers both LXR−SREBP1c activation and minor intron retention, thereby robustly upregulating *Srebp1c* expression at both the mRNA and protein levels.

MASLD is a progressive liver disease featured with early simple steatosis and late-stage severe hepatic fibrosis, and its pathogenesis is tightly controlled by stage-specific regulatory networks. Our results support that ZRSR1 and ZRSR2 are differentially regulated by distinct pathological signals throughout MASLD development, leading to disparate expression dynamics and biological roles. Our data reveal that hepatic ZRSR1 is selectively repressed in the early steatotic phase, and ZRSR1 deficiency alone is adequate to aggravate hepatic lipid deposition. By contrast, both ZRSR1 and ZRSR2 are markedly downregulated at advanced fibrotic stages and cooperatively promote liver fibrogenesis. Such temporal expression patterns and functional divergence between ZRSR1 and ZRSR2 may account for the stepwise progression of MASLD from simple steatosis to overt liver fibrosis (Fig. 7).

Collectively, these results highlight that correcting the impaired expression of minor intron splicing factors during early-stage MASLD could represent a promising therapeutic strategy for this disease.

### Limitations of the study

While ZRSR1 robustly inhibits LXR−SREBP1c-driven hepatic *de novo* lipogenesis, it exhibits no protective effect against hepatic inflammation. This uncoupled phenotype likely stems from its minimal influence on the synthesis of toxic lipid species including ceramides, which in turn limits its full therapeutic utility for clinical MASLD intervention. Furthermore, the upstream mole­cular signaling cascade responsible for HFD-mediated repression of hepatic ZRSR1 remains uncharacterized. In this work, *Zrsr1* expression was solely modulated via liver-tropic AAV vectors; we did not identify small-molecule ZRSR1 agonists or establish clini­cally translatable delivery modalities, leaving a notable translational gap between our mechanistic preclinical findings and viable therapeutic candidates for MASLD treatment.

## Materials and methods

### Animal studies

All animal studies were conducted in compliance with protocols reviewed and approved by the Institutional Animal Care and Use Committee of Shanghai Jiao Tong University (Approval number: JUMC2023-108-A). WT C57BL/6J mice were purchased from Shanghai Lingchang Biotechnology. All animals were housed under a standard 12-h light/12-h dark cycle with *ad libitum* access to food and water. Mice were fed regular chow diet, HFD (D12492, Research Diets), or CDA-HFD (MASH diet; A06071309, Research Diets). HFD feeding lasted for 13 weeks in *Zrsr1*-overexpressing mice and 7 weeks in *Zrsr1*-knockout mice, while WT mice were maintained on the MASH diet for 6 months. Hepatic *Zrsr1*-overexpressing mice and controls were generated by injecting AAV expressing *Zrsr1* (TBG-*Zrsr1*) or vector control (TBG-Vec) into WT mice, respectively. To generate liver-specific *Zrsr1* knockout mice and controls, liver-specific Cas9 transgenic mice were injected with AAV expressing *Zrsr1*-targeting sgRNA (*Zrsr1*-KO) or vector control (Ctr). Briefly, a total of 100 µL of AAV liquid containing 1 × 10^11^ viral genomes (vg) was injected via tail vein for each mouse. Mice were randomly assigned to treated or control groups, and investigators were blinded to genotype and treatment during data acquisition and analysis. All mouse experiments were independently replicated for at least twice. For LXR activation, mice were daily treated with T0901317 (Selleck, S7076) dissolved in sunflower oil via oral gavage at a dose of 25 mg/kg or vehicle (sunflower oil) for consecutive 4 days for *Zrsr1*-overexpressing mice and 3 days for *Zrsr1*-knockout mice. For histology, tissues were fixed overnight in 4% neutral buffered formalin at 4°C overnight, followed by paraffin embedding, sectioning, and H&E staining. Fresh liver samples were embedded in optimal cutting temperature compound, sectioned, and stained with ORO. Sirius Red staining was performed as described ­previously [29].

### Cell culture

Primary mouse hepatocytes were isolated from C57BL/6J mice by collagenase type II perfusion as reported [30]. Hepatocytes were maintained in Dulbecco’s Modified Eagle Medium (DMEM) supplemented with 10% fetal bovine serum (FBS) and 1% penicillin-streptomycin at 37°C in a humidified 5% CO_2_ incubator. Adenovirus infection was performed in the same day of isolation. After 24 h, cells were treated with vehicle or T0901317 (5 µmol/L) for another 24 h. The murine hepatocyte line AML12 (American Type Culture Collection) was cultured in DMEM/F12 medium (1:1, v/v) supplemented with 10% FBS, 1% penicillin-streptomycin, and ITS-G supplement. HEK 293 T cells were grown in DMEM containing 10% newborn bovine serum and 1% penicillin-streptomycin. All cellular experiments were conducted with triplicate samples and independently repeated at least three times.

### Metabolic analyses

For GTT, mice were fasted overnight (16 h) and then injected intraperitoneally (i.p.) with a glucose solution at 1.0 g/kg body weight. ITT was performed on mice fasted for 4 h prior to i.p. injection of insulin (1.0 U/kg body weight). Blood glucose concentrations were monitored at 0, 20, 45, 90, and 120 min post-injection using a glucometer. Plasma insulin concentration was determined using a commercially available ELISA kit (CrystalChem, 90095). In addition, standardized enzymatic assays were employed to determine plasma levels of NEFA, alanine aminotransferase (ALT), aspartate aminotransferase (AST), total triglyceride (TG), and total cholesterol (TC).

### Gene expression analyses

Mouse livers were immediately frozen in liquid nitrogen. Total RNA was extracted from tissue or cells using TRIzol reagent according to the manufacturer’s protocol. For cDNA synthesis, 2 µg total RNA was reverse-transcribed using HiScript II Q RT SuperMix (Vazyme, R222-01). The qPCR was performed with SYBR Green master mix (Vazyme, Q311) on Roche 480 light cycler. Relative mRNA level was normalized to the expression of *AP0*. All primers are listed in Supplementary Table S1.

### Immunoblotting analysis

Mouse liver tissue was excised and immediately frozen in liquid nitrogen. Tissue lysates were prepared by homogenizing in a buffer containing 50 mmol/L Tris-HCl (pH 7.6), 130 mmol/L NaCl, 5 mmol/L NaF, 25 mmol/L β-glycerophosphoate, 1 mmol/L sodium orthovanadate, 10% glycerol, 1% Triton X-100, 1 mmol/L DTT, 1 mmol/L PMSF, and a protease inhibitor cocktail. Total cell lysates were prepared in a lysis buffer containing 50 mmol/L Tris-HCl (pH 7.8), 137 mmol/L NaCl, 10 mmol/L NaF, 1 mmol/L EDTA, 1% Triton X-100, 10% glycerol, and a protease inhibitor cocktail (Bimake, B14002) after three freeze/thaw cycles. The antibodies used were as follows: ZRSR1 was customized from PTMbio; ZRSR2 (Invitrogen, PA5-41797) was from Thermo Fisher; SREBP-1 (sc-13551) and LXR (sc-377260) were from Santa Cruz; HSP90 (13171-1-AP), HA (51064-2-AP), FLAG (20543-1-AP), LAMIN A/C(10298-1-AP), and NF-κB p65 (65107451-1-AP) were from Proteintech. Phospho-AMPK (2535S), AMPK (2532), and Phospho-NF-κB p65 (3033) were from Cell Signaling Technology.

### RNA-seq data analysis

RNA-seq libraries were prepared from total liver RNA. Raw sequencing reads (fastq format) underwent quality assessment with FastQC and were aligned to the mouse reference genome (mm39/GRCm39) using HISAT2. Read counts per gene and for U12-type minor intron regions were generated with HTSeq [31]. Differential gene expression analysis was performed using the DESeq2 package in R [32]. Genes with an adjusted *P* value < 0.05 and an absolute fold change > 1.5 were considered significantly altered.

### *In vitro* lipogenesis assay

AML12 cells were incubated in Krebs Ringer buffer supplemented with 0.1% fatty acid-free BSA. Each well received 0.2 µCi/mL [U-^14^C]-acetic acid and incubated for 2 h with or without T0901317. The cells were lysed, and lipids were extracted using a hexane–isopropanol solution (hexane: isopropanol (v:v) = 3:2). The organic phase was collected, evaporated, and resuspended in toluene, and radioactivity was quantified using a liquid scintillation counter.

### Cut & Tag assay

Cut & Tag assay was performed using a commercialized kit (Vazyme, TD903) according to the manufacturer’s instructions. For each sample, pre-washed 1 × 10^5^ HA-LXR-overexpressing AML12 cells infected with adenovirus expressing GFP (Ad-GFP) or ZRSR1 (Ad-*Zrsr1*) in 100 μL wash buffer were incubated with 10 μL pre-activated ConA beads for 10 min at room temperature (RT) with gentle mixing. The supernatant was removed, and bead-bound cells were incubated with HA-tag antibody (1:50 dilution, Proteintech, 51064-2-AP) in 50 μL antibody buffer at 4°C overnight. Bead-bound cells were incubated with 50 μL of 1:100 unconjugated secondary antibody in Dig wash buffer for 1 h at RT with rotation, washed three times with 200 μL Dig wash buffer, and incubated with 0.04 µmol/L pA/G Tnp in Dig-300 buffer for 1 h at RT with rotation. After three washes with 200 µL Dig-300 buffer, cells were tagmented with 40 μL Dig-300 buffer supplemented with 10 μL Trueprep Tagment Buffer L (TTBL) for 1 h at 37°C. DNA fragmentation was stopped and DNA was extracted by adding 5 μL Proteinase K, 100 μL Buffer L/B, and 20 μL DNA Extract Beads, followed by vortexing and 10-min incubation at 55°C with two to three gentle mixing steps. Beads were washed once with 200 μL WA buffer and twice with 200 μL WB buffer. Fragmented DNA was eluted in 22 μL DNase-free water; 15 μL was used for library amplification according to the manufacturer’s instructions (Vazyme, TD202). Amplified DNA was purified with 100 μL VAHTS DNA Clean Beads (Vazyme, N411), eluted in 22 μL DNase-free water, and subjected to sequencing.

### Luciferase reporter assay

The *Srebp1c* promoters were amplified by PCR and cloned into the pGL3 Basic vector. AML12 cells were cultured in 24-well plates and transiently transfected with *Srebp1c*-luc (50 ng/well), LXRα (100 ng/well), and RXRα (100 ng/well), in combination with different doses of adenoviral *Zrsr1*. Cells were harvested for measuring luciferase activity after 36-h transfection. All reporter assays were repeated at least three times in triplicate.

### Hepatic triglyceride/cholesterol extraction and analyses

For quantification of hepatic triglyceride and cholesterol, tissue was homogenized with 1 mL buffer containing 300 mmol/L mannitol, 5 mmol/L EDTA, 50 mmol/L Tris-HCl (PH 7.5), and 1 mmol/L PMSF. Total lipids were extracted with 10 mmol/L KOH and chloroform/methanol (2:1, v/v). The mixture was vortexed vigorously and centrifuged. The bottom layer was transferred to a new tube, and chloroform/methanol/H_2_O (3:48:47, v/v/v) was added. The samples were mixed vigorously and centrifuged. The bottom liquid phase was transferred to a clean tube and vacuum-dried. Triglyceride and cholesterol were dissolved in butanol/[(Triton X-114/MeOH) (2:1, v/v)] (3:2, v/v). The triglyce­ride and cholesterol levels were measured using triglyceride and cholesterol assay kits, respectively, and normalized to the tissue weight.

### Co-IP analysis

HEK293T cells were co-transfected with the indicated plasmids. After 48 h, cells were lysed in RIPA buffer containing 150 mmol/L NaCl, 1% NP-40, 50 mmol/L Tris-HCl (pH 8.0), 25 mmol/L NaF, 2 mmol/L Na_3_VO_4_, 1 mmol/L PMSF, 1 mmol/L DTT, and a protease inhibitor cocktail. Lysates were sonicated briefly and then ­centrifuged. For each immunoprecipitation (IP), 2 µg of indicated antibody was added to the lysate and incubated overnight at 4°C. Protein A/G magnetic beads (Bimake, B23201) were then added and incubated for another 2 h. Beads were washed five to six times with cold wash buffer and proteins were eluted in 80 μL of 1× SDS loading buffer by boiling, followed by immunoblot analysis.

### Immunofluorescence

HEK293T cells were co-transfected with the indicated plasmids for 48 h and washed with PBS. Cells were fixed with 3.7% formaldehyde for 10 min, treated with 0.1% Triton X-100 (BBI, A600198) for 5 min, blocked with 3% BSA for 2 h at RT, and incubated with primary antibodies overnight at 4°C and with corresponding secondary antibodies for 2 h at the second day, and DAPI for 5 min at 37°C. The antibodies used were HA (Proteintech, 51064-2-AP, 1:100), FLAG (Proteintech, 20543-1-AP, 1:800), CoraLite594-conjugated Goat anti Rabbit IgG (H + L) (Proteintech, SA00013-4), and CoraLite488-conjugated Goat anti Mouse IgG (H + L) (Proteintech, SA00013-1). Images were captured using confocal microscope (Leica, SP8).

### Statistical analysis

Statistical analysis was performed using GraphPad Prism 9. Statistical differences were evaluated using two-tailed unpaired Student’s *t*-test for comparisons between two groups, or analy­sis of variance (ANOVA) and appropriate *post hoc* analyses for comparisons of more than two groups. Two-way ANOVA with multiple comparisons was used for statistical analysis of GTT, ITT, and body weight studies. A *P* value < 0.05 (*P < 0.05, **P < 0.01, and ***P < 0.001) was considered statistically significant. Statistical methods and corresponding *P* values for data shown in each panel are included in the figure legends.

## Supplementary Material

loag020_Supplementary_Data

## Data Availability

RNA-seq data files have been deposited at the Gene Expression Omnibus with accession number GSE315806.
