# Supplementary material for: ZRSR1 protects against obesity-associated hepatic lipogenesis independent of minor intron splicing
Source: Life Metab. 2026 Jul 8;5(5):loag020. doi: 10.1093/lifemeta/loag020 (PMC13439704; doi:10.1093/lifemeta/loag020)
Supplement: Supplementary file 1 [file loag020_supplementary_data.docx]

**Supplementary Information**

**Supplementary method**

**Cycloheximide (CHX) chase assays**

AML12 cells were infected with adenovirus expressing GFP (Ad-GFP) or *Zrsr1* (Ad-*Zrsr1*) for 36 h, and was treated with cycloheximide (CHX, 20 μg/mL) for variable durations (0, 15, 30, 60, and 120 min). Cells were collected and lysed with RIPA buffer. LXRα protein levels were analyzed by immunoblotting.

**Supplementary Figures**

**Supplementary Figure S1** HFD feeding induces obesity and hepatic steatosis without triggering fibrosis. (a) Body weight of mice fed a chow diet (*n* = 4) or a high-fat diet (HFD, *n* = 4) for 4 months. (b and c) Plasma (b) and liver (c) triglyceride (TG) levels of chow diet and HFD mice. (d) Hematoxylin and eosin (H&E) staining, Sirius Red staining of livers from chow diet and HFD mice (scale bar = 200 μm). Data are presented as the mean ± SEM. **P* < 0.05; *** *P* < 0.001, by two-tailed unpaired Student’s *t*-test (a-c); ns, not significant.

**Supplementary Figure S2** Hepatic *Zrsr1* overexpression and knockout do not alter basal lipid metabolism or minor intron splicing in chow diet-fed mice. (a and b) The qPCR (a) and immunoblotting (b) analyses of *Zrsr1* expression in the livers of AAV-TBG-Vec (TBG-Vec, *n* = 8) and AAV-TBG-*Zrsr1* (TBG-*Zrsr1*, *n* = 8) mice fed a chow diet. The relative amounts of ZRSR1 in b (left panel) were determined by the density of ZRSR1 bands, normalized to that of HSP90. Then, the fold changes were calculated by dividing the relative protein density of livers from TBG-Zrsr1 mice by that from TBG-Vec mice (b, right panel). (c) Immunoblotting analyses of ZRSR1 in the livers of liver-specific Cas9 transgenic mice injected with AAV expressing *Zrsr1*-targeting sgRNA (*Zrsr1*-KO, *n* = 5) or vector control (Ctr, *n* = 5) fed a chow diet. The relative amounts of ZRSR1 (left panel) were determined by the density of ZRSR1 bands, normalized to that of HSP90; Then, the fold changes were calculated by dividing the relative ZRSR1 density of livers from hepatic *Zrsr1*-KO mice by that from controls (right panel). (d) Fold change in minor (U12) intron reads from RNA sequencing data of livers in the TBG-Vec and TBG-*Zrsr1* mice (left panel), and in the hepatic *Zrsr1*-KO mice and controls (right panel). (e and f) Body weight and liver weight (as percentages of body weight) of TBG-Vec and TBG-*Zrsr1* mice (e), and of hepatic *Zrsr1*-KO mice and controls (f) fed a chow diet. (g) H&E staining of livers from TBG-Vec and TBG-*Zrsr1* mice (left panel), and from hepatic *Zrsr1*-KO mice and controls (right panel) fed a chow diet (scale bar = 100 μm). (h and i) Plasma NEFA, TG, and TC contents of TBG-Vec and TBG-*Zrsr1* mice fed a chow diet (h), and of hepatic *Zrsr1*-KO mice and controls fed a chow diet (i). (j) The food intake of AAV-TBG-Vec (TBG-Vec, *n* = 8) and AAV-TBG-*Zrsr1* (TBG-*Zrsr1*, *n* = 8) mice (left panel), and of *Zrsr1*-KO (*n* = 5) or Ctr (*n* = 5) mice (right panel) fed an HFD. Data are presented as the mean ± SEM. ****P* < 0.001, by two-tailed unpaired Student’s *t*-test (a,right panel of b and c, d−f, and h−j); ns, not significant.

**Supplementary Figure S3** ZRSR1 attenuates hepatic *de novo* lipogenesis in primary hepatocytes. (a−d) The qPCR analysis of lipogenic genes in primary hepatocytes infected with Vec- or *Zrsr1*-expressing adenovirus and then treated with vehicle (Veh) or T1317 (5 µmol/L) for 24 h. Data are presented as the mean ± SEM. ****P* < 0.001, by two-tailed unpaired Student’s *t*-test (a−d); ns, not significant.

**Supplementary Figure S4** ZRSR1 does not affect the LXR stability in AML12 Cells. (a) Immunoblotting analysis of LXR and ZRSR1 protein levels in AML12 cells infected with Ad-GFP or Ad-Zrsr1 for 36 h, followed by treatment with cycloheximide (CHX, 20 μg/mL) for different time points. (b) Quantification of LXR protein levels shown in (a). LXR band intensities were normalized to HSP90, and the relative LXR level at each time point was expressed as the fold change relative to the corresponding untreated group (0 h). Data are presented as the mean ± SEM, by two-way ANOVA with multiple comparisons (b); ns, not significant.

Supplementary Table S1 Primer sequences for qPCR.

| Primer | Forward (5’−3’) | Reverse (5’−3’) |
| --- | --- | --- |
| *Srebp1c* | ATCGGCGCGGAAGCTGTCGG | GGGAAGTCACTGTCTTGGTTG |
| *Srebp2* | TCAGCACCGCTCCGCAGACGAG | TACCGTCTGCACCTGCTGCTGG |
| *Chrebp* | GGGACAAGATCCGGCTGAA | GCTCTTCCTCCGTTGCACAT |
| *Scd1* | GCTGGAGTACGTCTGGAGGAA | TCCCGAAGAGGCAGGTGTAG |
| *Fasn* | GGTTACACTGTGCTAGGTGTTG | TCCAGGCGCATGAGGCTCAGC |
| *Dgat2* | GCGCTACTTCCGAGACTACTT | GGGCCTTATGCCAGGAAACT |
| *Ap0* | GAAACTGCTGCCTCACATCCG | GCTGGCACAGTGACCTCACACG |
| *Fsp27* | TCGACCTGTACAAGCTGAACCCT | AGGTGCCAAGCAGCATGTGACC |
| *Zrsr1* | CGGTGGAAGGTTGCAATTTG | CACCGCTACGGCGGACGCTACGGC |
| *Zrsr2* | GCTCGCTCGGCTCAGAGA | TTGTGCCTTTTCTTCCCTCAG |
| *Ccl2* | aggtccctgtcatgcttctg | tctggacccattccttcttg |
| *Ccl5* | TGCCCACGTCAAGGAGTATTT | TTCTCTGGGTTGGCACACACT |
| *Il6* | agttgccttcttgggactga | tccacgatttcccagagaac |
| *Il1β* | GAAATGCCACCTTTTGACAGTG | TGGATGCTCTCATCAGGACAG |
| *Hmgcs2* | GACATCAACTCCCTGTGCCTG | GATGTCAGTGTTGCCTGAATC |
| *Ehhadh* | CAGATGAAGCACTCAAGCTTG | ACCTTGGCAATGGCTTCTGCA |
| *Relb* | CCGAATCAACAAGGAGAGCG | TGTGCTGAACACCACGGATA |
| *TNFα* | CAGGCGGTGCCTATGTCTC | CGATCACCCCGAAGTTCAGTAG |
| *Pparα* | GCAGTGCCCTGAACATCGA | CGCCGAAAGAAGCCCTTAC |
| *Ppary* | GAAAGACAACGGACAAATCACC | GGGGGTGATATGTTTGAACTTG |
| *Lxrα* | AGGCTCAAGCCACTTCGGTGTC | AGAAGGAGCGCCTGTTACACTG |
| WT-*Insig1* | CTGTATTGCCGTGTTCGTTG | CTTCGGGAACGATCAAATGT |
| WT-*Insig2* | agagtggtccagtgtcatgc | cagccagtgtgagggaaaac |
| U12-*Insig1* | TTTCTGTGCTACGTCCAGAGT | GGGTAGGTACCACCATCATGT |
| U12-*Insig2* | AAGGACCTTGAGGGAGTTGC | TTGCAACGAAGCCATTTCCC |
